# Combined accelerometer and genetic analysis to differentiate essential tremor from Parkinson’s disease

**DOI:** 10.7717/peerj.5308

**Published:** 2018-07-20

**Authors:** Bhuvan Molparia, Brian N. Schrader, Eli Cohen, Jennifer L. Wagner, Sandeep R. Gupta, Sherrie Gould, Nelson Hwynn, Emily G. Spencer, Ali Torkamani

**Affiliations:** 1 The Scripps Translational Science Institute, The Scripps Research Institute, La Jolla, CA, USA; 2 Department of Integrative Structural and Computational Biology, The Scripps Research Institute, La Jolla, CA, USA; 3 Intel Corporation, Santa Clara, CA, USA; 4 Scripps Clinic Torrey Pines, Scripps Health, La Jolla, CA, USA

**Keywords:** Accelerometer, Movement disorder, Tremor, Genetic risk, Genetic risk score, Essential tremor, Polygenic risk score, Parkinson’s disease

## Abstract

Essential tremor (ET) and Parkinson’s disease (PD) are among the most common adult-onset tremor disorders. Clinical and pathological studies suggest that misdiagnosis of PD for ET, and vice versa, occur in anywhere from 15% to 35% of cases. Complex diagnostic procedures, such as dopamine transporter imaging, can be powerful diagnostic aids but are lengthy and expensive procedures that are not widely available. Preliminary studies suggest that monitoring of tremor characteristics with consumer grade accelerometer devices could be a more accessible approach to the discrimination of PD from ET, but these studies have been performed in well-controlled clinical settings requiring multiple maneuvers and oversight from clinical or research staff, and thus may not be representative of at-home monitoring in the community setting. Therefore, we set out to determine whether discrimination of PD vs. ET diagnosis could be achieved by monitoring research subject movements at home using consumer grade devices, and whether discrimination could be improved with the addition of genetic profiling of the type that is readily available through direct-to-consumer genetic testing services. Forty subjects with PD and 27 patients with ET were genetically profiled and had their movements characterized three-times a day for two weeks through a simple procedure meant to induce rest tremors. We found that tremor characteristics could be used to predict diagnosis status (sensitivity = 76%, specificity = 65%, area under the curve (AUC) = 0.75), but that the addition of genetic risk information, via a PD polygenic risk score, did not improve discriminatory power (sensitivity = 80%, specificity = 65%, AUC = 0.73).

## Introduction

Essential tremor (ET) and Parkinson’s disease (PD) are among the most common adult-onset tremor disorders. The prevalence of ET and PD in individuals who are 65 years of age and older is ∼4.5% and ∼2.0%, respectively (De Rijk et al., 2000; Louis & Ferreira, 2010). Clinical differentiation of ET from PD can be challenging, especially in the early stages of disease where clinical signs are subtle and often overlap (Jain, Lo & Louis, 2006; Tolosa, Wenning & Poewe, 2006). Clinical and pathological studies suggest misdiagnosis of PD in patients with other movement disorders ranges anywhere from 15% to 35%, with a diagnosis of PD when the true diagnosis is ET, or vice versa, being the most common misdiagnoses (Hughes et al., 1992; Hughes, Daniel & Lees, 2001; Jain, Lo & Louis, 2006; Litvan et al., 1998; Rajput, Rozdilsky & Rajput, 1991; Schrag, Ben-Shlomo & Quinn, 2002; Tolosa, Wenning & Poewe, 2006). Complex diagnostic procedures performed by specialists, such as surface electromyography, electroencephalography, and dopamine transporter imaging, can be powerful diagnostic aids but are not often performed because they are lengthy and expensive procedures that are not broadly available (Antonini et al., 2008; Thenganatt & Louis, 2012). Thus, the clinical differentiation of these movement disorders often relies on responsiveness to levodopa-challenge and the presence of cardinal symptoms including, but not limited to, bradykinesia, rigidity, and the presence of resting vs. kinetic or postural tremors, especially limb tremors (Tolosa, Wenning & Poewe, 2006).

The most common and classical PD tremor is a four to six Hz resting limb tremor, whereas the most common ET tremor is a four to eight Hz postural or kinetic tremor (Chen et al., 2017). However, both resting and kinetic tremors can be observed in both PD and ET, with resting tremors having been reported in up to 46% of individuals with a diagnosis of ET and postural tremors having been reported in up to 90% of individuals with a diagnosis of PD (Louis, Hernandez & Michalec, 2015; Thenganatt & Louis, 2012), though the reported range of this overlap in tremor types ranges widely across studies. Detailed clinical examination with attention to specific tremor features (frequency, amplitude, pattern, and distribution) and associated neurological findings can help distinguish patients with the two diseases (Thenganatt & Louis, 2012). However, due to the similarities in tremor characteristics for PD vs. ET, distinguishing some of these features without electronic aids can be difficult (Chen et al., 2017; Jain, Lo & Louis, 2006; Thenganatt & Louis, 2012).

A number of initial studies suggest that objective monitoring of tremor features with an accelerometer can be utilized to extract subtle characteristics that distinguish PD from ET (Barrantes et al., 2017; Brennan et al., 2002; Burne et al., 2002; Deuschl et al., 2000; Henderson et al., 1994; Hossen et al., 2013; LeMoyne et al., 2010, 2015; Woods et al., 2014; Zeuner et al., 2003; Zimmermann et al., 1994), however the vast majority of these studies utilized accelerometer monitoring in conjunction with more sophisticated surface electromyography and/or clinical evaluations. Two studies suggest that consumer grade accelerometers, that is, those embedded in modern smartphones, can be utilized to distinguish between PD and ET diagnosis in a well-controlled clinical setting (Barrantes et al., 2017; Woods et al., 2014). While the results of these prior studies are highly encouraging, achieving between 85% and 95% accuracy, testing either required maneuvers administered by support staff, and/or was performed in a selected population with a confirmed pathological hand tremor. While these studies are of great interest, and perhaps represent aspirational targets for discriminative performance, the underlying data is not likely to be representative of the noisy data that would be collected during a home monitoring session administered solely by the patient. Thus, we set out to determine whether discrimination of PD vs ET diagnosis could be achieved by monitoring research subject movements at home with a basic testing procedure using consumer grade devices.

In addition, we examined whether the inclusion of genetic information of the type that is widely available via direct-to-consumer genetic testing services could improve discriminatory power and potentially compensate for the increased heterogeneity of tremor characteristics that would be expected in an unselected patient population monitored at home. PD, like many common diseases, is often thought of as having two genetic forms; an early-onset form characterized by a strong family history and inherited in a monogenic fashion, and a late-onset form that is often referred to as “sporadic” and inherited in a polygenic fashion (Klein & Westenberger, 2012). Approximately 5–10% of PD is due to the monogenic form of the disease, which can be readily recognized due to family history, age-of-onset, and disease presentation features indicative of specific gene mutations (Lesage & Brice, 2009). Genetic testing for monogenic disease is not necessary to reduce diagnostic uncertainty, but is suggested to help better inform disease prognosis and prioritization of treatment (Klein & Westenberger, 2012). It is largely the more common, late-onset, sporadic form of PD that is associated with diagnostic uncertainty and the subject of this study. The heritability of sporadic PD, due to polygenic factors, is estimated at 25–30% (Do et al., 2011; Keller et al., 2012), suggesting that polygenic risk scores could eventually be useful for identifying a portion of individuals at risk for PD. Importantly, genetic risk is largely established at birth, and thus could potentially be an effective tool for predicting PD status at early disease stages when clinical signs are subtle. While only <10% of the heritability of PD has been explained by current genetic findings (Darweesh et al., 2016; Nalls et al., 2014; Pihlstrom et al., 2016), and while the clinical utility of genetic testing for PD outside of early onset patient populations is limited (Klein & Westenberger, 2012), genetic risk models have been shown to predict PD status from healthy controls, or correlate with aspects of PD severity or progression, with modest but statistically significant accuracy (Hall et al., 2013). In addition, it is not known whether PD and ET share genetic risk factors that may interfere with genetic differentiation of the two conditions, nor how PD genetic factors interact with movement features to potentially improve discriminatory power. In this light, we performed a pilot-scale study to determine whether combined accelerometer and genetic analysis has the potential to differentiate the diagnosis of essential tremor from Parkinson’s disease.

## Methods

### Study recruitment and consent

Study participants provided written informed consent under the protocol entitled “The Genetic and Digital Diagnosis of Essential Tremor and Parkinson’s Disease” (GADGET-PD), which was approved by the Scripps Institutional Review Board in 2015 (IRB-15-6628). The trial is also listed on ClinicalTrials.gov (NCT02668835).

Recruitment for GADGET-PD was done through a neurology clinic within the Scripps Health system and through an emailed flyer distributed by the International Essential Tremor Foundation to its members in the San Diego, CA area. Inclusion criteria for the study were an established clinical diagnosis of PD or ET, ability to provide informed consent, and willingness to have a blood draw. Exclusion criteria included: (1) presence of dementia, (2) presence of other known neurological disorders besides PD or ET, or (3) history of bone marrow transplant. Pharmacological or surgical treatments of tremor were not exclusion factors. Diagnoses were established by the Unified Parkinson Disease Rating Scale and the American Academy of Neurology guidelines for PD and ET, respectively. The diagnosis of idiopathic Parkinson’s disease was confirmed by a movement disorders specialist utilizing the UK Brain Bank criteria. Fourty subjects with PD and 27 patients with ET were recruited for the study.

### Limb movement monitoring

Each participant was provided with an LG G2 smartphone pre-loaded with the Fox Insight mobile application which is part of Intel® Pharma Analytics Platform, modified to launch a cognitive attention task, and a Pebble Smart-watch. Mobile phones and Pebble watches were paired via Bluetooth by our research coordinator during orientation of the participants. Once paired, the watch displayed a persistent notification: “Fox Insight Wear,” signifying successful pairing. If connection was lost, the notification changed to “Disconnected.” Participants were asked to wear the smart-watch on their tremor dominant hand, and keep the smartphone in their possession, at all waking hours of the day for a period of two weeks to avoid any disconnections. Participants were also given the contact information of our research coordinator, and IT helpdesk to assist with repairing of devices in case of any failures. Since, some of our participants were older and not very tech savvy, we also monitored their daily gameplays, and contacted them if we noticed missing data for consecutive 2 to 3 days.

Participants were asked to perform a cognitive attention task, which also initiated the collection of raw accelerometer data, three times a day; (1) upon waking in the morning prior to consuming medication, (2) in the afternoon 1 h after consuming medication, or at 1 PM if not medicated, and (3) in the evening, immediately prior to sleep. To perform the cognitive attention task and initiate accelerometer data collection, participants were asked to:

- Sit in a natural seated position; upright on a raised seat of standard height with both feet resting on the floor.
- Place their tremor dominant hand with the smart-watch device on their lap, resting and fully supported against gravity.
- Hold the smartphone device in their other hand with the phone parallel to the floor and screen fully visible.
- Open the Fox Insight application on the smartphone and initiate the cognitive attention task.
- All data collection for the movement monitoring was done by the Intel® Pharma Analytics Platform which later made all data available for data analysis.

Cognitive attention tasks, especially those including a motor task, have been shown to provoke motor impairment and tremors in the non-attended limb (Fleminger, 1992; Song et al., 2010; Woods et al., 2014). In this case, the cognitive attention task consisted of attending to the hand holding the smartphone and playing a logic game—*tiltmazes* (https://code.google.com/archive/p/tiltmazes/)—for 2 min. In *tiltmazes* a ball sits in a flat tray containing a maze with one or more square goals. The challenge is to guide the ball through the maze and collect all the squares. This process ensures normalization of the position of the body and arms during time the movement data is collected, normalizes duration of the movement data collection itself across subjects, minimizes the movements required by the participant to complete the cognitive attention task while maintaining the attention of the participant, and engages the participant in a cognitive attention task (hereafter referred to as a “gameplay session”) that is interesting enough to be repeated thrice daily for a span of two weeks. By popular demand, the *tiltmazes* game was provided to research subjects outside of the confines of the Fox Insight application.

At the end of the 2 min gameplay session, a short tremor questionnaire administered by the smartphone asked the following questions:

- Did you experience any tremors while playing the game? (yes/no)
- Which hand experienced tremors? (phone/watch/both/none)

### Movement data processing

The raw accelerometer data was sampled every 20 milliseconds (50 Hz). Each gameplay session was designed to last 2 min, but we noticed some gameplay sessions which went well beyond the 2-min recording mark, and others which were ended prior to the 2-min mark. Thus, we first filtered to include only gameplay sessions where the participant reported experiencing a tremor on either hand. In order to account for problems with data recording, stemming from device connectivity issues, delays in answering the tremor questionnaire, etc., the span of each gameplay session was defined as the period of time, starting from up to 5 min prior to the response to the tremor questionnaire, containing consecutive accelerometer data points recorded within at least 1 s of each other. It should be noted that detailed tremor data is not available outside of gameplay sessions, thus non-gameplay recordings would not influence the results.

Next, each data point from the raw movement data, which was initially recorded by the smart-watch device as triaxial acceleration values (mGs, 1G = 9.8 m/s^2^), was converted to an overall magnitude of acceleration per data point by calculating the root of sum of squares, resulting in a vector of acceleration values per gameplay session. Each vector of acceleration values was passed through a Butterworth band-pass filter to retain only movements in the three to seven Hz frequency range. Finally, the total energy, average amplitude, and maximum amplitude value for each filtered gameplay session was calculated.

### Genetic data collection and polygenic risk score calculation

Genotyping of blood samples from each research subject was performed using the Affymetrix UK Biobank Axiom Array following standard manufacturer instructions (Affymetrix Inc., Santa Clara, CA, USA). A PD polygenic risk score was calculated using a weighted allele-counting approach (Dudbridge, 2013) based on known, common PD susceptibility loci, derived from the largest and most recent PD genome-wide association study (GWAS) meta-analysis (Nalls et al., 2014). Twenty two SNPs identified as genome-wide significant in the discovery stage and replicated in the validation stage of this GWAS meta-analysis were retained. When the index SNP reported in this GWAS was not directly genotyped on the UK Biobank Axiom Array, the most highly correlated SNP present on the array was used as a proxy. See Table S1 for the full list of SNPs, proxies, and odds ratios used to calculate the PD PRS.

### Data analyses and code availability

Data processing was performed with custom code written in python. Code is available at the Torkamani Lab github repository (https://github.com/TorkamaniLab/). Genetic data is not available for distribution. All statistical analyses were performed in R (R Core Team, 2008). In the linear models, PD affection was coded as the positive outcome.

## Results

### Movement analysis

**Figure 1:**
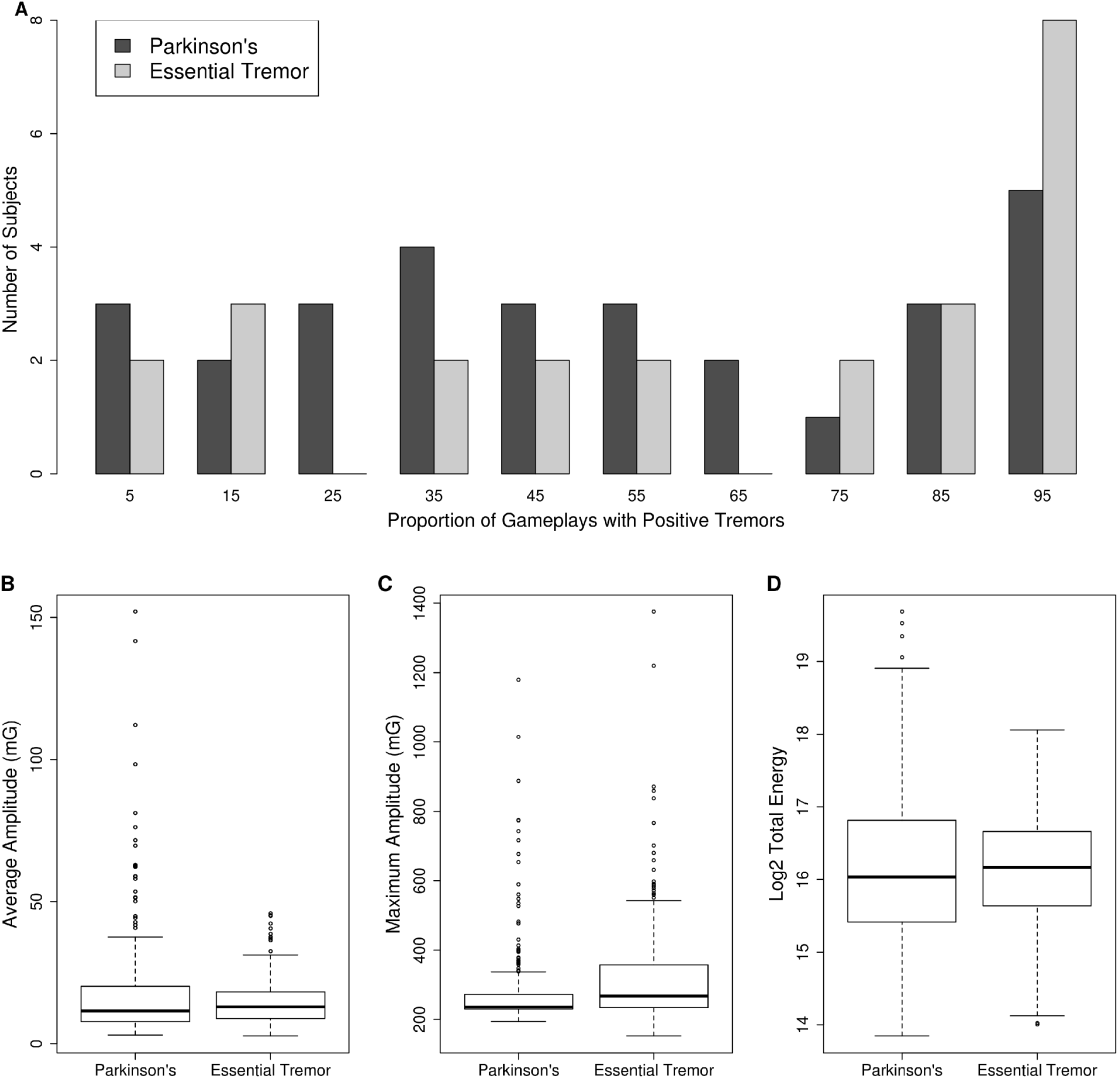
Tremor monitoring characteristics. (A) The distribution of the proportion of gameplay sessions where research subjects reported experiencing a tremor is presented as a histogram. PD = dark-gray bars, ET = light-gray bars. Note research subjects reported experiencing tremors across the entire range of proportion of gameplays. The average amplitude (milli Gs, 1G = 9.8 m/s^2^) (B), maximum amplitude (milli Gs, 1G = 9.8 m/s^2^) (C), and total energy (D) of three to seven Hz movements recorded during gameplay is plotted as a boxplot for PD vs ET. The box represents the interquartile range (25th to 75th percentile) and the whiskers represent 1.5 times the interquartile range. Individual data points outside this range are plotted as open circles.

The basic demographic characteristics, time since diagnosis and medication status of the PD and ET subjects is presented in Table 1. Initially, 40 subjects with PD and 27 patients with ET were recruited for the study. Movement data collection failed for three ET subjects and seven PD subjects leaving a final cohort of 33 subjects with a diagnosis of PD and 24 subjects with a diagnosis of ET. For all remaining subjects at least one gameplay session associated with a participant reported tremor was recorded. The distribution of the proportion of gameplay sessions where the participant reported experiencing a tremor is plotted in Fig. 1A. On average, ET subjects reported experiencing a tremor in 63% (SD = 34%) of gameplay session, whereas PD subjects reported experiencing a tremor in 52% (SD = 31%) of gameplay sessions. This difference was not statistically significant (*t*-test *p*-value = 0.5 after log transformation of proportions) as both PD and ET subjects experienced tremors during gameplay sessions at a broad range of rates, from 2% to 100% of gameplay sessions in both PD and ET subjects (Fig. 1A).

**Table 1:** Research subjects’ characteristics.

Characteristic	Parkinson’s disease	Essential tremor
Number of subjects	40	27
Gender	Male = 24 (60%)	Male = 13 (48%)
	Female = 16 (40%)	Female = 14 (52%)
Median age (SD, Range)	73 years (±8.4, 46–90)	73 years (±10.6, 34–90)
Median time since diagnosis (SD, Range)	3 years (±2.6, 1–13)	9 years (±13.5, 1–50)
Medicated to treat PD or ET?	33 (83%)	18 (66.7%)

Next, we compared the total energy, average amplitude, and maximum amplitude of movements in the three to seven Hz frequency range across PD and ET subjects. The distribution of these measures is provided in Figs. 1B–1D. Maximum amplitude was significantly different between PD and ET subjects. The maximum amplitude was 280 (SD = 123) for PD vs 328 (SD = 158) for ET (*p*-value = 1.7 × 10^−8^, Mann–Whitney *U* test) (Fig. 1C). Whereas both the average amplitude (Fig. 1B) and total energy of movements (Fig. 1D) were not significantly different between PD and ET subjects. The average amplitude was 18.0 (SD = 19.3) for PD vs 14.8 (SD = 8.3) for ET (*p*-value = 0.375, Mann–Whitney *U* test) while the log_2_ total energy was 16.18 (SD = 1.1) for PD vs 16.13 (SD = 0.82) for ET (*p*-value = 0.59, Mann–Whitney *U* test).

### Genetic analysis

**Figure 2:**
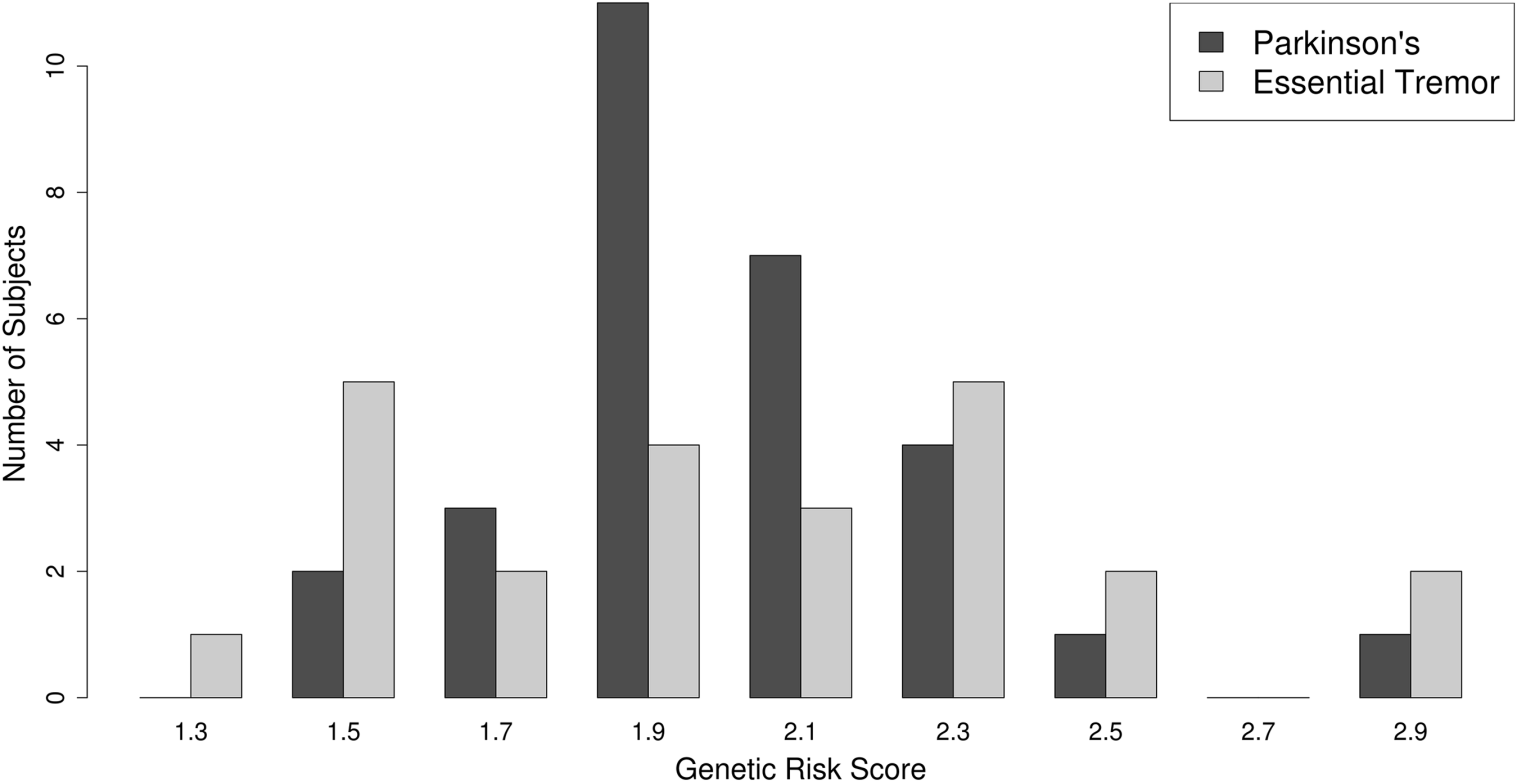
Polygenic risk score distribution. The distribution of the PD polygenic risk score is presented as a histogram. PD = dark-gray bars, ET = light-gray bars.

As described in “Methods,” a PD polygenic risk score was calculated using a weighted allele-counting approach (Dudbridge, 2013) based on 22 known, common PD susceptibility loci, derived from the largest and most recent PD GWAS meta-analysis (Nalls et al., 2014). The distribution of the PD polygenic risk scores is plotted in Fig. 2. No difference was observed between the PD PRS scores achieved by PD or ET subjects. The average PD polygenic risk score value was 2.00 (SD = 0.3) SD for PD vs 2.01 (SD = 0.4) for ET (*p*-value = 0.965, Mann–Whitney *U* test).

### Combined model

**Figure 3:**
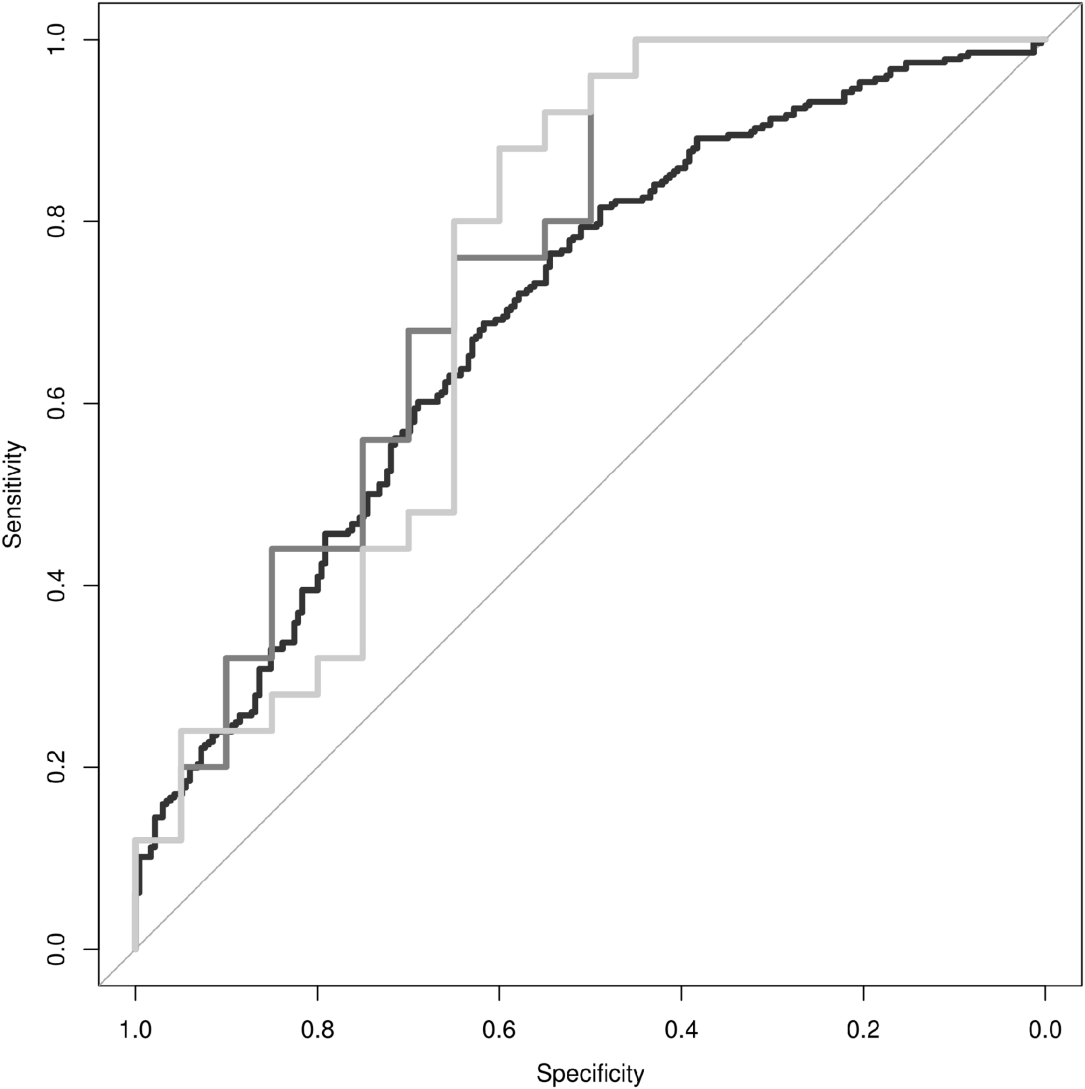
Discrimination of PD from ET. Receiver operating characteristic curves are provided for movement data based classification of individual gameplay sessions (black line, AUC = 0.65), classification of individual research subjects based on the median classification of their respective individual gameplay sessions (dark-gray line, AUC = 0.72), and classification of individual research subjects based on the combination of their movement data and polygenic risk score (light-gray line, AUC = 0.70).

Finally, we sought to determine whether discrimination of PD from ET could be achieved using movement data features combined with a PD polygenic risk score. First, we designed a simple linear model, where each gameplay session was treated independently, to determine if the linear combination of log_2_ total energy, average amplitude, and maximum amplitude could predict whether the gameplay session was derived from a subject diagnosed with PD vs. ET. The linear model was able to predict the diagnosis status of the individual associated with each individual gameplay session with an adjusted *R*^2^ = 0.05 (*p*-value = 6.0 × 10^−6^). The receiver operating characteristic (ROC) curve for this linear model is presented in Fig. 3 (black line), each individual gameplay session could be classified as coming from a PD or ET subject with a modest area under the curve (AUC) of AUC = 0.696. Both average amplitude (β = 0.187, *p*-value = 4.5 × 10^−6^) and maximum amplitude (β = −0.136, *p*-value = 4.17 × 10^−8^) were independently significant predictors of diagnosis status.

In order to combine individual gameplay predictions into a summary prediction of diagnosis status per subject, we utilized the median prediction of each subject’s gameplay sessions as the summary prediction of their diagnosis status. The ROC curve for this linear model is presented in Fig. 3 (dark-gray line). This summary predictor was able to differentiate PD from ET subjects accurately with a sensitivity of 76%, specificity of 65%, and AUC = 0.75.

Finally, we generated a linear model to differentiate PD from ET subjects by utilizing the median prediction of gameplay sessions per individual plus their polygenic risk score. This model was able to differentiate PD from ET accurately with a sensitivity of 80%, specificity of 65%, and AUC = 0.73 (Fig. 3, light-gray line). The median prediction from movement characteristics was significantly predictive of diagnosis status (β = 2.98, *p*-value = 0.003), however, while the addition of the polygenic risk score did improve the maximum sensitivity and specificity values of the model, it did not significantly improve discrimination of PD from ET overall (β = −0.07, *p*-value = 0.72).

## Discussion

The purpose of this study was to determine whether easily accessible genetic information and movement features, measured through consumer grade devices and services, could be utilized as a means to differentiate PD from ET, either to supplement current diagnostic approaches or provide an alternative diagnostic path for individuals in circumstances where access to specialized tests and movement disorder experts may be limited. This study represents, to the best of our knowledge, the first attempt to combine genetic profiling with digital monitoring to improve differential diagnosis of two common and related conditions. One could imagine a scenario in the future where early detection and classification of these movement disorders is accomplished through the passive and automatic monitoring of these features prior to any overt symptoms or suspicion of disease onset (Torkamani et al., 2017). While this is the ultimate goal, passive monitoring introduces additional challenges with data heterogeneity—challenges which significantly impacted the discriminatory performance of our study relative to prior studies where data collection was performed under the supervision of research staff. We found that the diagnosis of PD could be distinguished from ET with modest accuracy by monitoring rest tremor characteristics induced by a cognitive attention task, measured at home using a consumer grade accelerometer, and following a simple procedure requiring no specific maneuvers or oversight by clinical staff. It should be noted that the accuracy of classification based on tremor features could be improved by more advanced signal processing techniques, however, we sought to determine whether the combination of genetic plus movement characteristics could provide complementary information rather than determine the maximal extent of the predictive power derived from any individual predictive feature. In this light, we found that the addition of polygenic risk information did not significantly improve discrimination of PD from ET.

While polygenic risk information appears to be unlikely to contribute significantly to the differentiation of PD from ET, there are a number of caveats and limitations of this study that may have limited our ability to detect a genetic signal. First, a relatively small proportion (<10%) of the heritability of PD has been explained by current GWAS findings (Darweesh et al., 2016; Nalls et al., 2014; Pihlstrom et al., 2016), thus it is possible that more complete knowledge of common PD genetic risk factors could provide a predictive signature. Similarly, given the relatively small sample size of this study, a significant predictive genetic signal could have been detected in a larger study, though the discriminatory power of that signal would likely be weak. Second, the genetic information utilized here did not include familial PD genetic risk variants, though this class of genetic risk variant is known to contribute minimally to the incidence of PD (Klein & Westenberger, 2012). Third, the genetic components of ET were not included in this analysis, given that no truly genome-wide significant risk loci have been detected (Muller et al., 2016). Finally, it is possible that misdiagnosis of ET vs PD in our cohort itself obscures any true genetic signal. While our subjects were clinically diagnosed according to accepted international standards, the premise of this study is based on the observation that those standards can result in misdiagnoses (Hughes et al., 1992; Hughes, Daniel & Lees, 2001; Jain, Lo & Louis, 2006; Litvan et al., 1998; Rajput, Rozdilsky & Rajput, 1991; Schrag, Ben-Shlomo & Quinn, 2002; Tolosa, Wenning & Poewe, 2006). Misdiagnosis in living individuals may introduce a fundamental limitation of differential diagnosis for these diseases, or at least represent a major challenge to the development of improved diagnostic procedures. Definitive diagnosis can only be achieved through post-mortem neuropathological assessment, which obviously is not possible for this study (Dickson et al., 2009). Interestingly, all ET subjects reported experiencing a rest tremor during this study, which is a higher rate than expected given the cardinal signs of PD vs ET differ in tremor type (Chen et al., 2017). However, it is possible that this observation is due to the reporting of physiological tremors rather than true rest tremors. Similarly, observation of our research participants during the informed consent and brief training period process suggests that research subjects underreported the incidence of tremors when sufficiently distracted by the cognitive attention task, leading to clearly visible tremors that went unnoticed by the participants themselves.

## Conclusions

In summary, we show that common direct-to-consumer devices used to monitor movement can be useful for distinguishing common tremor disorders, and can possibly be extended to identify first onset of tremor disorders in a continuous monitoring setting. We further found that while genetic profiling may be useful for the identification of individuals with early-onset PD due to familial genetic risk variants, it is unlikely that polygenic risk information contributes substantially to the differentiation of the more common forms of sporadic PD from ET. Regardless, larger studies utilizing more complete genetic knowledge of PD and ET genetic risk factors may have the potential to improve discrimination in the future.

## Supplemental Information

10.7717/peerj.5308/supp-1 Supplemental Information 1 Parkinson’s risk SNPs and odds ratios.

## Additional Information and Declarations

### Data Availability

The following information was supplied regarding data availability: Genetic data is not available for distribution because study participants did not consent to genetic data redistribution. Detailed data analysis methods, and relevant results are available on GitHub: https://github.com/bmolparia/pd_vs_et.
